# Data-Monitoring Solution for Desalination Processes: Cooling Tower and Mechanical Vapor Compression Hybrid System

**DOI:** 10.3390/s24092909

**Published:** 2024-05-02

**Authors:** Paula Hernández-Baño, Angel Molina-García, Francisco Vera-García

**Affiliations:** 1 Department of Automatics, Electrical Engineering and Electronic Technology, Universidad Politécnica de Cartagena, 30202 Cartagena, Spain; paula.hernandez@upct.es; 2 Department of Thermal Engineering and Fluids, Universidad Politécnica de Cartagena, 30202 Cartagena, Spain; francisco.vera@upct.es

**Keywords:** hybrid systems, water desalination, real-time remote monitoring process, sensor

## Abstract

The advancement of novel water treatment technologies requires the implementation of both accurate data measurement and recording processes. These procedures are essential for acquiring results and conducting thorough analyses to enhance operational efficiency. In addition, accurate sensor data facilitate precise control over chemical treatment dosages, ensuring optimal water quality and corrosion inhibition while minimizing chemical usage and associated costs. Under this framework, this paper describes the sensoring and monitoring solution for a hybrid system based on a cooling tower (CT) connected to mechanical vapor compression (MVC) equipment for desalination and brine concentration purposes. Sensors connected to the data commercial logger solution, Almemo 2890-9, are also discussed in detail such as temperature, relative humidity, pressure, flow rate, etc. The monitoring system allows remote control of the MVC based on a server, GateManager, and TightVNC. In this way, the proposed solution provides remote access to the hybrid system, being able to visualize gathered data in real time. A case study located in Cartagena (Spain) is used to assess the proposed solution. Collected data from temperature transmitters, pneumatic valves, level sensors, and power demand are included and discussed in the paper. These variables allow a subsequent forecasting process to estimate brine concentration values. Different sample times are included in this paper to minimize the collected data from the hybrid system within suitable operation conditions. This solution is suitable to be applied to other desalination processes and locations.

## 1. Introduction

The escalation of global warming and climate change has pushed water scarcity to a critical threshold. According to estimates, 34 countries are grappling with water stress levels ranging from 25% to 75%, while 25 nations, including Iran, are facing stress levels exceeding 75%. Projections from the World Health Organization suggest that by as early as 2025, approximately half of the global population will reside in water-stressed regions [1]. Furthermore, the escalating demand for water has led to a decline in freshwater availability. Since the 1980s, water demand has steadily increased by around 1% annually, and it is anticipated to surge from current consumption levels by 20% to 30% by 2050 [2]. Concurrently, global energy consumption is on an upward trajectory, projected to surge by nearly 50% by 2035 and 80% by 2050 [3], further straining energy resources. In parallel, the escalating global water scarcity has spurred increased reliance on groundwater to fulfill freshwater requirements. Despite the prevalence of brackish conditions in most groundwater reservoirs, with total dissolved solids (TDS) ranging from 500 mg/L to 5000 mg/L, [4], brackish groundwater (BGW) remains largely underutilized in numerous regions worldwide. Enhanced utilization of brackish groundwater holds potential to alleviate escalating strain on freshwater resources, especially in arid, inland areas. Different authors agree that desalination technologies can effectively lower the salinity levels in brackish groundwater, rendering it suitable for various freshwater applications [5]. Desalination technologies in commercial use can be categorized into two primary groups: membrane-based and thermal processes. Membrane processes, such as reverse osmosis (RO) and electrodialysis (ED), utilize semipermeable membranes to selectively allow or block the passage of specific salt ions. Thermal methods replicate the natural evaporation–condensation cycle by heating saline water to produce vapor, subsequently condensed into fresh water. Nevertheless, both commercially available RO and thermal desalination using electricity or fossil fuels are energy-intensive. Moreover, thermal desalination methods powered by electricity or fossil fuels are known for their high energy consumption [6]. As a result, there is growing interest in integrating renewable energy sources (RES) into desalination processes. Promoting hybrid systems that combine various RES is essential for achieving optimal efficiency under similar operating conditions. They are regarded as a cost-effective solution offering multiple benefits, including reduced specific energy consumption, lower overall system cost, and increased plant efficiency leading to maximum output. The hybrid desalination sector is experiencing a surge in innovative developments, reflecting its growing importance in this field [7]. On the other hand, recent authors affirmed that utilizing saline groundwater (SGW) as a feedstock for desalination offers several advantages, including reduced maintenance requirements and lower energy consumption compared to seawater, rendering it economically appealing [8,9,10]. Actually, the required energy to produce fresh water varies according to different water sources: from 0.48 kWh/m^3^ groundwater, to 2.5–8.5 kWh/m^3^ seawater [11].

Multi-effect desalination systems (MED) powered by mechanical vapor compression (MVC) are attractive systems for seawater/brackish water desalination due to the fact that these systems are independent of steam generation sources and hence fit well in remote areas [12]. In addition, MED-MVC desalination plants can directly be driven by renewable energy-based sources like photovoltaic (PV) solar systems or wind/PV hybrid systems [13], thereby reducing CO_2_ emissions as well. A recent stand-alone system of a modified multi-effect mechanical vapor compression (ME-MVC) desalination unit powered by photovoltaic/thermal-photovoltaic (PVT-PV) collectors was discussed in [14]. The designed system was modeled and solved using MATLAB in terms of economic and energy impacts. Results provided that the combined subsystem gave the lowest electricity consumption (6.39 kWh/m^3^), while the value for SE-MVC was 7.58 kWh/m^3^. In addition, Koroneos et al. [15] affirmed that the most attractive feature of the MVC system was that it only required either electrical or mechanical energy as the primary energy input, being significantly small compared to the potential energy of steam. Field data showed that the MVC demanded about 10–14 kWh/m^3^, including the pumping and compressor power [16]. Nevertheless, Zejli et al. [17] concluded that future work should be focused on monitoring both electrical and thermal demand to the current water demand as a suitable way to study an optimal control strategy more adequate to real-time control and efficiency monitoring.

Repurposing discharged cooling tower water within the industry reduces the industrial demand for freshwater and helps to ease the strain on natural freshwater reservoirs. In a cooling tower, a portion of the cooling water evaporates to remove heat [18]. Furthermore, only a fraction of the steam utilized for process hot utility is reclaimed as condensate return. As a result of evaporation within the cooling tower, the salt concentration in the cooling tower water rises [19]. Marazgioui et al. [20] affirmed that cooling towers play a crucial role in dissipating heat in concentrated solar power (CSP) plants. Indeed, the effectiveness of CSP plants largely hinges on the selection of cooling tower technology. This study seeks to evaluate how different cooling tower technologies affect CSP plants in terms of performance, economics, and environmental considerations. Cooling towers have also contributed significantly to industrial freshwater extraction, and repurposing the effluents from cooling tower water (CTW) can significantly reduce industrial freshwater footprints [21].

Avila-Filho et al. [22] affirmed that it is imperative to recognize the significance of enhancing the precision of information pertaining to cooling towers. Particular emphasis must be placed on the instrumentation utilized in controlling tower and cooling system operations. This is crucial in averting erroneous decisions regarding the withdrawal and dissipation of thermal loads into the atmosphere. Consequently, the adoption of precise measuring instrumentation or estimation methodologies becomes imperative to enhance the reliability of cooling control processes. Moreover, Hosoz et al. [23] emphasized that the thermal performance of cooling towers is usually determined experimentally, requiring the mathematical models to have mostly a large number of geometrical parameters to define the system, which may not be readily available. The specific literature provides reactive and predictive maintenance strategies to assess current operating conditions and forecasting potential malfunctioning of the cooling tower [24]. Yamaguchi et al. [25] constructed a portable system for rapid on-site Legionella monitoring in order to enumerate target bacterial cells, in line with other contributions [26,27]. Nevertheless, Tian-Hong et al. [28] noted that minor attention had been paid to the performance evaluation of a cooling tower during regular operation.

By considering these contributions, the present paper describes the sensoring and monitoring solution for a hybrid system based on a cooling tower connected to a mechanical vapor compression equipment for desalination and brine concentration purposes. This contribution is also in line with recent authors that pointed out the necessity of long-term field data for the large-scale pumping of SGW, being crucial to provide a better understanding of the hydrological system and verify hydrological models [29].

The rest of the paper is structured as follows. Section 2 describes the proposed monitoring solution for desalination processes based on a cooling tower and MVC hydrid system (CT-MVC). Section 3 provides the results and discussion about the proposed solution and the potential benefits from the results. Finally, Section 4 gives the conclusions.

## 2. Materials and Methods

The hybrid technology CT-MVC is analyzed to be implemented for zero liquid discharge (ZLD) processes. The high electrical consumption in the MVC system aims to conduct further study [12]. Combining diverse technologies is an upcoming strategy to reduce negative effects of the brine in desalination [30]. Previous works and analyses were carried out to design the set MVC-CT, being appropriate according to the operational conditions [31,32]. MVC works with higher salinity, avoiding deposits in the CT packing and pipes, being heat transfer-enhanced accordingly. In addition, ambient conditions have a significant role in the CT performance. MVC is a closed system, and this factor is mitigated. As previously mentioned, membrane desalination processes are defined for pre-concentrating brine. The CT-MVC methodology can thus treat higher conductivity in a second step, reaching a ZLD system. The standalone CT and MVC can achieve a brine reduction of 50% and 46%, respectively. Otherwise, the hybrid technology is able to provide a higher percentage of 73%.

**Figure 1:**
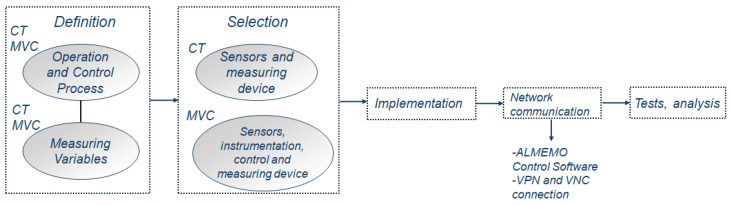
General task sequencing.

Monitoring in the water desalination process is widely spread as a result of the required control due to plants operating 24 h a day. Information in real time is received by operators and the response is immediate; hence, there is a lower risk of a severe breakdown or an unsatisfactory process over time. Recent technologies demand an accurate oversight, and this study presents the design, implementation, and performance evaluation of the monitoring in a hybrid system: CT-MVC for brine. Firstly, this approach is defined in Figure 1; to start, measuring values are specified, then, cooling tower sensors are selected with their data acquisition system, including the installation and configuration. Simultaneously, because of the complexity, the sensing, instrumentation, control and recording process of MVC is thoroughly carried out. In advance of the experimental procedure, the testing framework for management and remote connections is executed. As a result, the obtained experimental data lead to a comprehensive analysis.

### 2.1. Cooling Tower System Description

**Figure 2:**
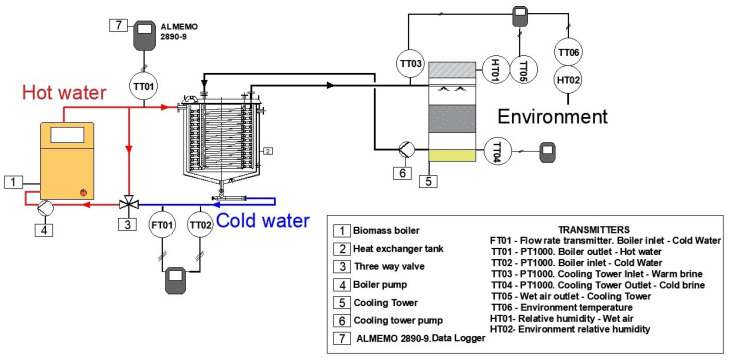
Cooling tower system scheme.

**Figure 3:**
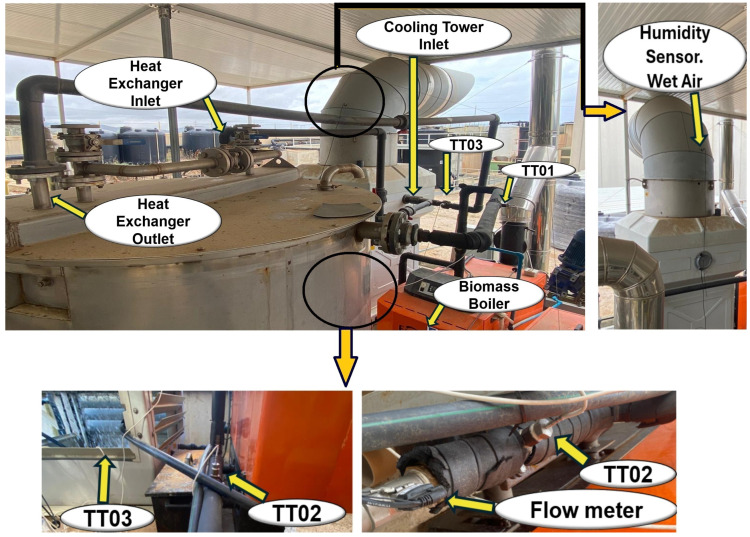
Monitoring, sensing and elements of the cooling tower. A general perspective.

A CT operates by circulating water through a shell and tube exchangers, where the heat extracted from the process is transferred to the cooling tower. The primary purpose is to lower the water temperature through heat and mass exchange with the ambient dry air propelled by fans. The mass balance of the system includes the release of water vapor due to evaporation, leading to a reduction in temperature as the air removes heat along with the evaporated water [33]. Therefore, a thermal performance analysis requires temperature and humidity measurements. A detailed diagram in Figure 2 describes the proposed CT monitoring solution. PT1000 sensors (Almeno–ZA9030) are selected to collect water temperature data in boiler as well as both brine currents. The deployed humidity/temperature sensors (Almeno-FHAD 46-C41AL05) provide the evaporation progress data and environment conditions. Physical variables are then gathered through these sensors that send the corresponding signal data to the selected Almeno 2890-9 data logger. Additionally, the boiler and heat exchange performance results are also monitored. With this aim, it is selected a flow meter to determine the water circulation from boiler to tank and conversely. Detailed features of the temperature, humidity, and flow rate sensors can be found in Table 1. A real representation of the cooling tower performance is illustrated in Figure 3.

The Ahlborn Almemo 2890-9 data logger displays and stores measured physical values, allowing a WLAN network communication. An Almemo© control freeware provides users a detailed configuration of the sensors and devices, being able to download or view data in real time. A host computer linked with Almemo receives information from it wirelessly using Wi-Fi. A remote desktop connection for external devices is a way to have knowledge about scenario evolution in distant environments from the local network. Technical data of Almemo 2890-9 are collected in Table 2. Recent applications to this data logger can be found in [34,35] for thermal systems also.

### 2.2. MVC System Description: Instrumentation and Functional Elements

MVC technology for desalination applications uses compressed steam to evaporate the inlet current of brine at vacuum pressure and lower temperature. The proposal method is analyzed and evaluated alongside membrane techniques and other thermal processes in previous stages. Seawater is commonly treated and concentrated by osmosis in a preliminary stage. The subsequent brine with high salinity cannot be fed into the membrane equipment, the evaporation being then considered a feasible way to increase freshwater recovery. Comprehensive contributions in the specific literature about desalination and brine treatment were studied to gain a comparative insight between emerging and traditional technologies, and further information can be found in [12,30,32,36,37,38,39,40,41,42].

**Figure 4:**
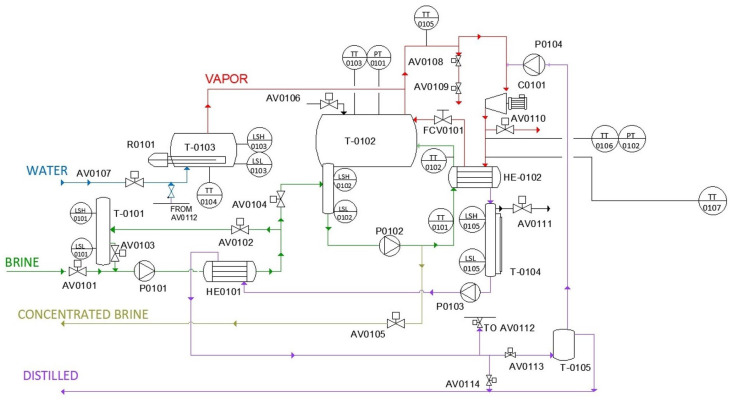
MVC scheme. From TT0101 to TT0107: Temperature sensor–transmitter. FCV0101: Valve for vapor recirculation. From AV0101 to AV0114: Other valves. T-0101: Brine feed tank. T0102: Flash tank. T-0103: Boiler. HE0101: Brine pre-heater. HE0102: Brine heater. C0101: compressor.

**Figure 5:**
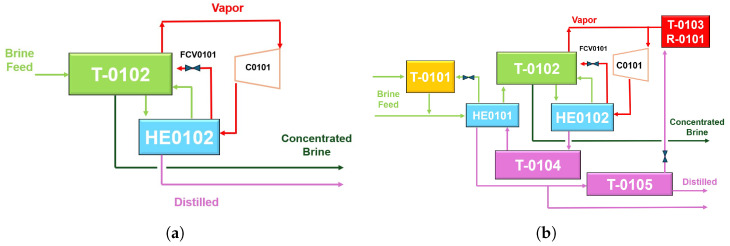
Simplified operational mode for MVC system. (**a**) Ideal performance. (**b**) Real performance.

**Figure 6:**
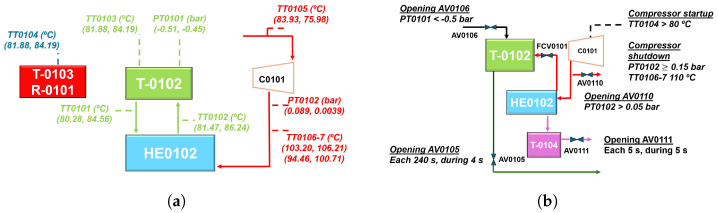
Simplified operational mode of MVC system. (**a**) Measuring points and physical variables. (**b**) Control points and actions.

The process in MVC equipment is automatically controlled by a programmable logic controller (PLC) with its robust instrumentation, to conduct thorough thermal analysis. The proposal scheme is represented in Figure 4. A detailed diagram of the ideal process can be found in Figure 5a. The system is autonomous in terms of heating energy. The generated and compressed vapor can raise the inlet brine temperature to keep evaporating new product. Figure 5b also depicts the flow of currents, brine supply, vapor, concentrated brine, and distilled. Measuring points and the relevant physical variables are represented in Figure 6a, considering an operational range for an ordinary day. Streams are shown in different colors due to salinity and physical state differences. Section 3 gives a comprehensive analysis of pressure and temperature monitored variables. The valve performance is summarized in Table 3 and illustrated in Figure 6b for a better comprehension.

**Figure 7:**
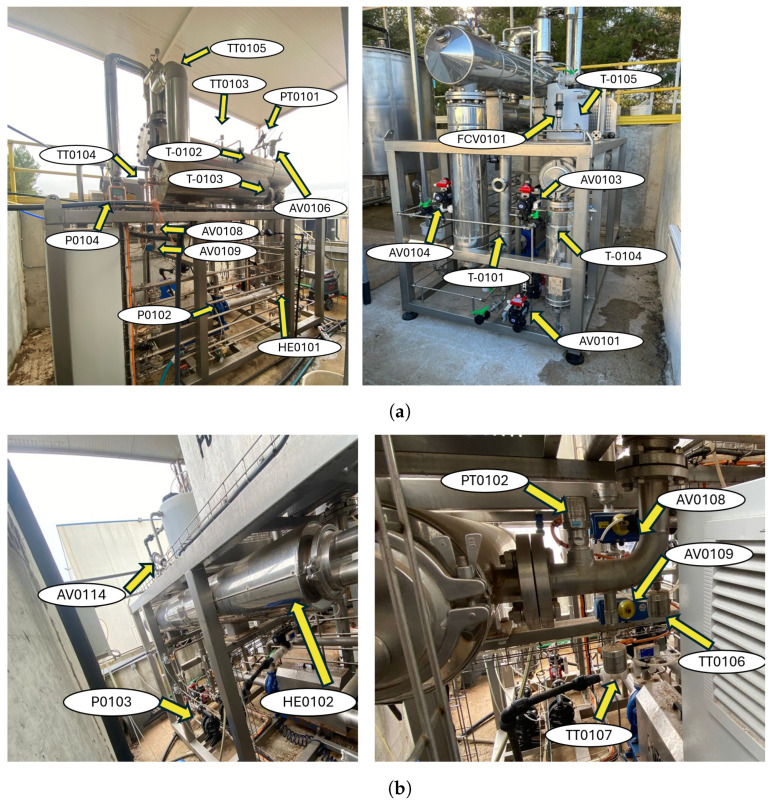
Case study for the proposed MVC solution. (**a**) Side and front view. (**b**) Side and detailed view.

The experimental operation was comprised of diverse stages that help to explain the designed model. In the first one, the system exhibits a transient behavior and the 13.50 kW TOPE resistance (R0101) is turned on all the time. The executed programming allows to start up the compressor at specific temperature of the TT0104 transmitter, in particular, 80 °C. Compressor activation is performed in a slope of 5 Hz, between 16 and 50 Hz. This initial phase ends when the configurable solenoid valve (AV0105) opens, and concentrated brine is discharged from the system, but TT0103 has to display 70 °C. Then, two steady states are recognized, and the treated brine flows out with a frequency of four minutes during four seconds. Understanding that the demand of thermal energy cannot be supplied by compressed vapor, exclusively, one stationary state is defined when the resistance is powered on. The other one considered happens while resistance is disconnected. For both, a set point of temperatures is tested and developed in Section 3. Moreover, the programmable FCV0101 pneumatic valve regulates the recirculation of compressed vapor in T0102 percentage-wise, depending on the heat device state. Principally, the utmost involvement is reached when R0101 is OFF. This behavior is analyzed and shown in Section 3. Independently, the brine feed does not affect to system temperature, and it solely relies on LMT102 high/low level sensors by IFM, which are installed in the reservoirs. Solenoid valves with pneumatic actuator from AV0101 to AV0104 receive a signal, and the flow into the MVC is produced. The prototype safety leads to a focus on the venting system. Relative pressure in T0102 is measured by PX3254 transmitter from IFM. Readouts are received by the control mechanism, and the AV0106 angle seat valve allows the ventilation flow if the relative pressure is less than −0.50 bar. Governing equipment stops the compressor when PT0102 measurements are higher than 0.15 bar. To avoid this over-pressure, valve AV0110 opens when the pressure is 0.050 bar. Additionally, volatile particles are removed from the distillate tank (T0104) through another pneumatic valve AV0111, programmed to open and close for 5 s. Finally, two configurable valves AV0108 and AV0109 automatically remove the condensates from the compressor with 5 min time interval. Figure 7 depicts both elements and sensing equipment.

### 2.3. MVC Control and Network Communications

**Figure 8:**
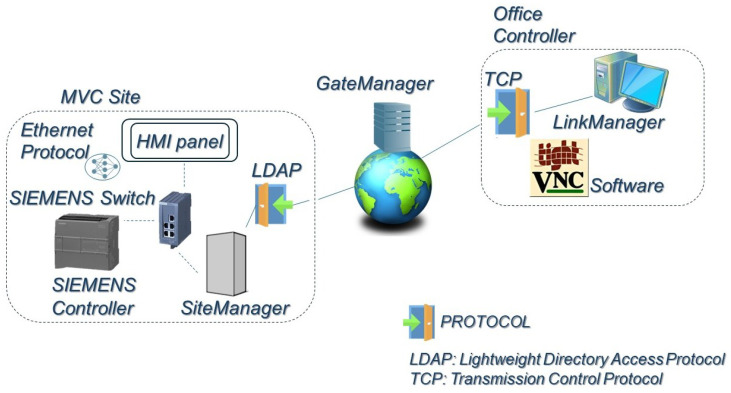
Network communication. Components of the MVC site: PLC, HMI Panel and SiteManager, attached by Ethernet Protocol and the SIEMENS Switch. GateManager server and the industrial environment are communicated by the LDAP Procotol. Office Controller is illustrated with two components, LinkManager 8 and VNC Software, to link with the MVC Site and establish remote connections.

The control and remote access are required to maintenance operations, mainly to avoid downtime for the operators, less time of acting, and lower the probability of equipment failures, representing an important element of Industry 4.0. The root of the automation in the MVC system is Siemens programmable logic controller (PLC) and the corresponding complementary modules. Communication in the industrial environment is carried out through the Scalance Switch by Ethernet protocol. The procedure scheme is shown in Figure 8. The PLC component is programmed to act in function of measuring values by sensors–transmitters. In the planning and configuring steps, the required sensors (level, temperature, and pressure), pumps, and valves are linked via PLC for an accurate operation.

**Figure 9:**
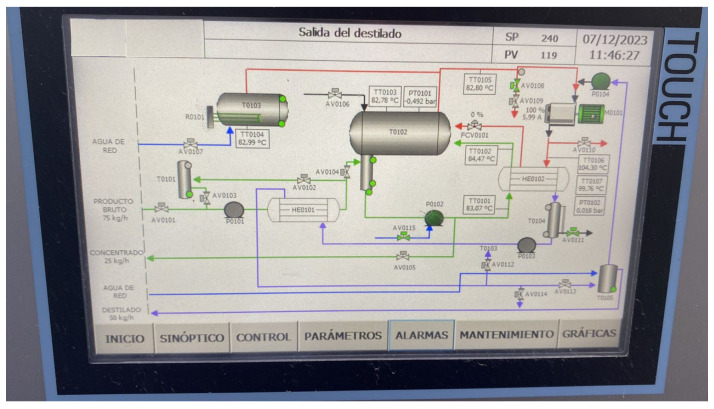
Example of SIMATIC human–machine–panel.

Parameters as brine feed depend on the transmitters’ high–low levels, resistance startup is performed in function of the water temperature, and pressure control is possible by automatic driven valves. Therefore, PLC implementation provides the developed working mode. In parallel, SIMATIC human–machine–panel (HMI) optimizes industrial process because of its intuitive graphical interface. The incorporated screen is showing in real time the equipment behavior as seen in Figure 9. The control is also executed from it, such as powering up vapor production, or stopping the treatment.

The external communication to the programmable controller and devices is managed by accessible software. The path to follow is represented in Figure 8. The first element in the connectivity framework from the industrial environment is SiteManager hardware, which is an internet of things (IoT) gateway similar to a VPN router for machines. The transmission protocol between SiteManager and GateManager is a lightweight directory access protocol (LDAP). GateManager controls and approves connections, and also manages gadgets. The software server is used at the administrator level to govern the oversight of the gateways to SiteManager. The users interact with the LinkManager interface and survey the available devices list for remote connections, which is created by the GateManager server through the TCP protocol. Finally, they can choose the desired networked equipment. In the study scenario, connection to HMI panel device starts from LinkManager, then, operators employ virtual network computing (VNC) for remote desktop support with the IP address. The advantage of disposing this platform is a higher supervision for the correct machinery access.

## 3. Results and Discussion

The hybrid technology MVC and CT worked and collected data for a month in Cartagena. The sophistication of MVC compelled the testing of the equipment for three months, independently. A comprehensive study of the proposal sensing and monitoring is presented based on the gathered data. Measured variables were saved each minute, and the operation of the elements (pumps, compressor, valves, etc.) is provided in real time for the MVC case. Initially, the cooling tower treats the brine, evaporating and concentrating about 50% in equal parts. The reject stream feeds the MVC from a connection valve in batches. As a result, the thermal and mechanical processes produce two currents, roughly, where 46% is distillate and the rest of the brine is more densely concentrated. The combined result of the hybrid system is 26% concentrated brine in relation to the total brine input.

### 3.1. Monitoring and Sensing Data Analysis of the Cooling Tower Solution

**Figure 10:**
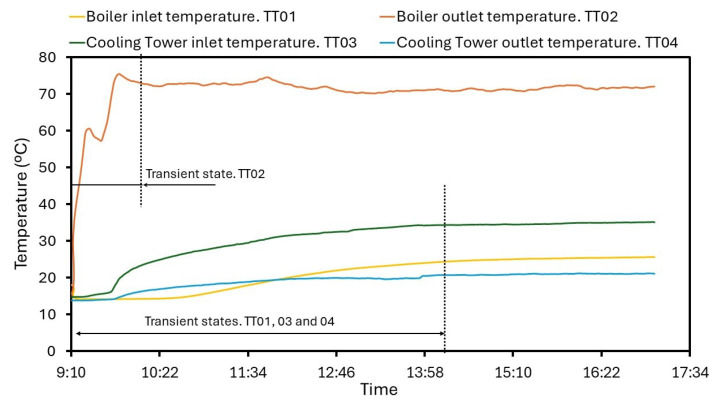
Temperature evolution for an operation day.

**Figure 11:**
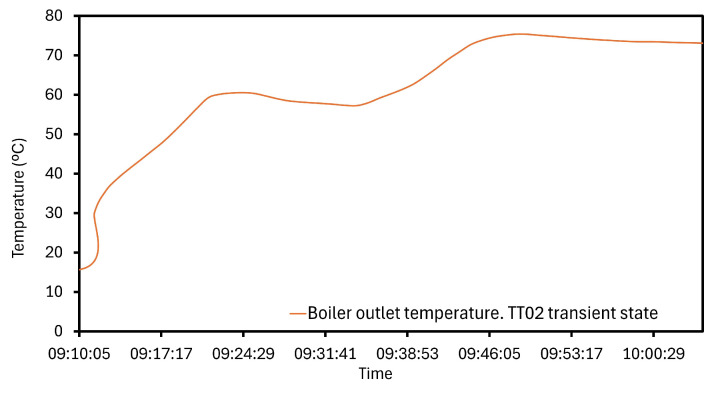
Increasing temperature at the boiler inlet. Transient state.

**Figure 12:**
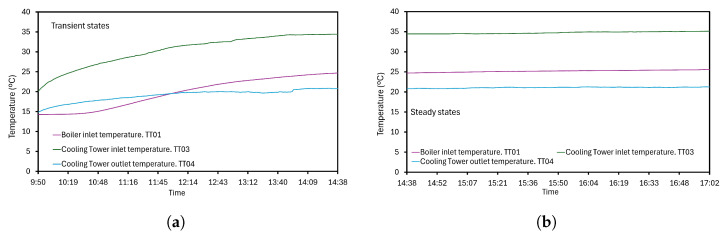
Temperature evolution: (**a**) Transient state. (**b**) Stationary state.

The sensing performance study is based on a heating test. This scenario covers a wide variation of the temperature range, and the analysis begins evaluating it. The main water from the biomass boiler and brine start to heat around 13.34 °C. PT1000 sensors located at critical points measure the represented values in Figure 10 over the operation. At the boiler outlet, TT02 deploys the highest values and a peak of 75.38 °C is found. However, TT02 remains stable around 70.24 °C when the steady period is reached. The sensor behavior in the transient state is represented in Figure 11 where the oscillations are more notable. In general, during the operation, the temperature rises to 56.70 °C, being registered by the PT1000 in a fast response time. Water at the boiler outlet has the lowest temperature, starting from 14.19 to 25.56 °C, and the maximum reading is 25.59 °C. As a result, these represented values in Figure 12 are the most linear. Heat supplied to the inlet brine causes a slow warming rising to 35.16 °C at the end of the operation. Despite the small increase which is observed during the study in Figure 10, the TT03 sensor is able to show insignificant fluctuations due to its sensitivity. Generally, these fluctuations are produced in terms of hundredths. The distribution of temperatures in Figure 10 also illustrates the cooling tower outlet. This variable depends on both the inlet temperature and the performance progress. The TT04 average value is 19.23 °C, and the maximum and minimum measured values are 21.26 °C and 13.34 °C, respectively.

Transient and steady states are shown in Figure 12 for the corresponding TT01, 3 and 4 sensors. Note small changes over time while temperature is increasing in Figure 12a. As the operation progresses, the TT03 device reduces its variability, developing an increase of 2.98 °C/h under the transient state. Figure 12b shows the stabilization of brine temperature, modifying its value only 0.29 °C/h. TT01 and 4 sensors provide the same behavior and, despite minor fluctuations at the CT outlet, the collected data of such variables are not identical but significantly similar. Therefore, measuring devices are suitable for a quick and accurate enough evaluation.

**Figure 13:**
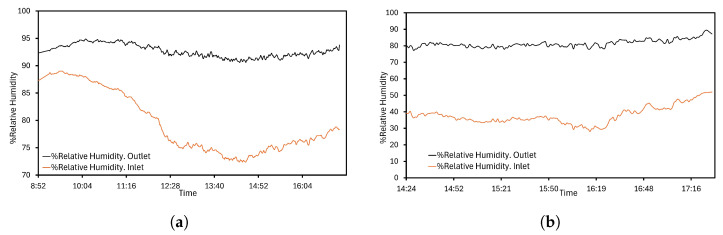
Relative humidity: (**a**) Operation with ambient wet air. (**b**) Operation with ambient dry air.

Relative humidity variable has a significant role in CT performance. An accurate measuring value is crucial to the evaluation procedure. The corresponding selected humidity sensors for the outlet wet air and ambient conditions are in charge of measuring and sending data to the acquisition system. Typically, desalinization processes are in locations next to the coast. Air has a high relative humidity, but sometimes it can be reduced. For this reason, a sensor with a broad range of measuring and tests is selected, assumed by the authors as being appropriate for this kind of application. The information is collected in Figure 13, where two scenarios of relative humidity are represented. In Figure 13a, air in contact with brine achieves values around 90%. The highest register is 94.90% and the minimum is 90.60%, while ambient shows humidity from 79.30 to 72.40%. Alternatively, other intervals were also studied. The operation with conditions of dry air is illustrated in Figure 13b. Collected data provide lower percentages: from 28.10% to 52.00%. The wet air outlet shows a working range of around 80% humidity, similar to the results given in Figure 13a.

**Figure 14:**
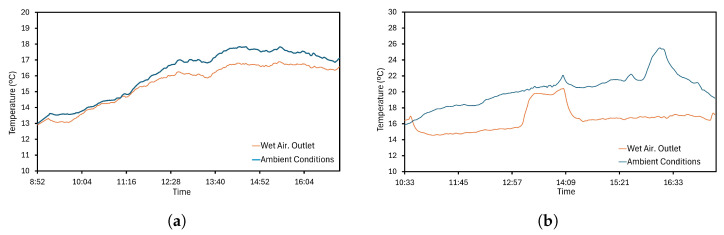
Air temperature: (**a**) Operation day 1. Temperatures of the wet air and ambient conditions. (**b**) Operation day 2. Temperatures of the wet air and ambient conditions.

The FHAD 46-C41AL05 humidity sensor can also measure temperature values. Conditions for wet air and ambient are represented in Figure 14. A growing trend is observed in Figure 14a from 13 °C to 17 °C with variability. The second setting in Figure 14b shows a greater difference between both collected data. Wet air has stable behavior until midway through the operation, where a sudden increase is observed. This unexpected behavior allows to detect undesired fan shutdowns, and preventive actions could be considered to improve the performance of the global system. From the collected data, similar intervals were identified during the season, but the tendency sensor proves an ability to determine higher temperatures, being that the maximum is 25.49 °C in Figure 14b.

**Figure 15:**
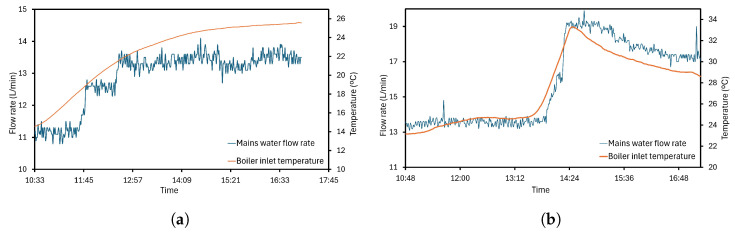
Evolution of flow rate depending on the inlet temperature. (**a**) Working with lower flow rate. (**b**) Working with higher flow rate.

According to the scheme previously depicted in Figure 2, the three-way valve has an important function for the main water flow rate. Only a mixture above 55 °C is able to enter the boiler, avoiding condensates. The installed flow meter can read data from 10 to 200 L/min, but the operation conditions enable checking the lower end of the range, following Figure 15. The fluctuations depending on the temperature are visible in Figure 15a: a 11.10 L/min flow rate is measured for 14.61 °C, being the minimum reading. As the operation progresses, an appreciable increase at the boiler temperature is accompanied by a flow rate variation between 12.70 and 14.10 L/min. These oscillations could be produced by inertial phenomena to which PT1000 does not exhibit any change. Also, other stream rates are shown for higher temperatures in Figure 15b, in this case dependence between the two variables is more evident. The registered peak is 19.90 L/min for 32.55 °C; on the contrary, the maximum temperature is 33.29 °C with 19.20 L/min of recirculated water.

### 3.2. Monitoring, Sensing and Control System Data Analysis of the MVC Solution

**Figure 16:**
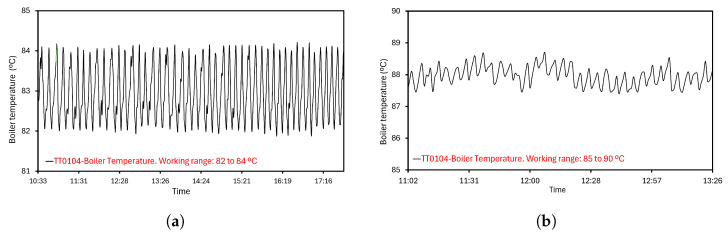
Programmed working ranges for the resistance. (**a**) Resistance working range from 82 to 84 °C. (**b**) Resistance working range from 85 to 90 °C.

The heat requirements are supplied by controlled R0101 resistance. Configuration of the temperature working range is essential to the evaporation and energy performance. The system is set up to govern this parameter, so the resistor control is executed, measured, and recorded. Two ranges are tested and shown in Figure 16. In the mode depicted in Figure 16a, a set point is fixed from 82 to 84 °C and verified by TT0104 measures. In Figure 16b, higher temperatures are established, 85 to 90 °C, and the TT0104 results indicate that resistance is ON all the time. Consequently, a greater energy consumption occurs, despite the evaporation performance being similar in both scenarios. This effect leads to the work in Figure 16a.

**Figure 17:**
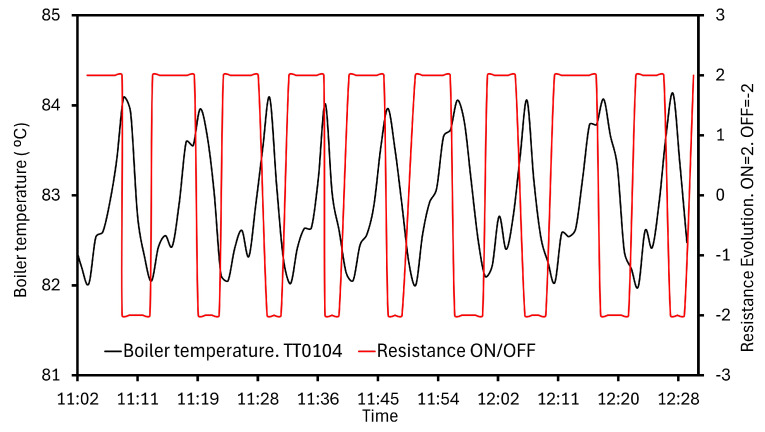
Resistance governance depending on the boiler temperature.

R0101 management is more clearly illustrated in Figure 17. High resistance values are sent and recorded by the system, and the correct information is ratified with the TT0104 measuring. When this state is happening, the boiler temperature must be increasing due to the thermal contribution, as it is shown in Figure 17. The collected temperatures may be slightly over 84 °C due to the inertia phenomena, but following the configuration, they are never above this value over time. On the other hand, a low level means that resistance is turned off and the boiler temperature trajectory reflects a decline. Reaching 82 °C, the system switches on the heating device, receiving the gathered measurements from TT0104.

**Figure 18:**
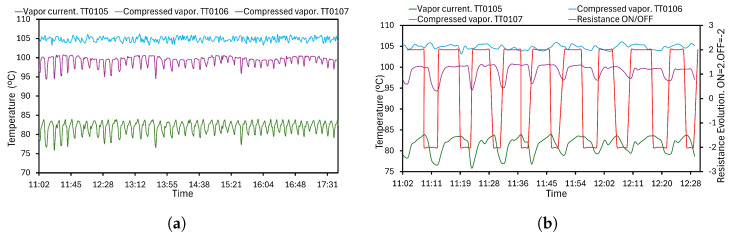
Vapor and compressed vapor temperatures. Variations with the resistance state. (**a**) Measurements from TT0105, 6 and 7 in an operation day. (**b**) Measurements from TT0105, 6 and 7 with the resistance values in a short period of operation.

In addition, the temperature estimation can be determined in other MVC locations to offer very thorough monitoring for future works. Vapor and compressed vapor data are determined by TT0105, TT0106, and TT0107 transmitters. The results for an eight-hour study are summarized in Figure 18a under steady-state conditions. The maximum and minimum temperature values for TT0105, 6 and 7 are 83.93–75.98 °C, 106.21–103.20 °C, and 100.72–94.47 °C, respectively. The observed fluctuations in Figure 18 are due to the resistance operation. The elaborate graph in Figure 18b for a short period can see the variations. Vapor and compressed vapor streams increase their temperatures while resistance is ON. As expected, this variable starts to drop with the resistance disconnection.

**Figure 19:**
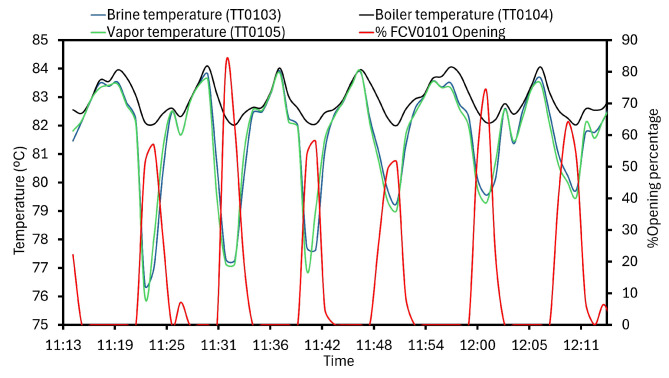
FCV0101 control and variations with the temperature.

The MVC is equipped with a controlled pneumatic valve FCV0101 that regulates the compressed vapor recirculation into T0102. The flow rate was programmed to be forwarded from FCV0101 in terms of percentage according to Figure 19. For the illustrated time frame, the maximum percentage of opening was 81.77%. At the same time, TT0103, 4, and 5 provided 77.31 °C, 82.28 °C, and 77.1412 °C, respectively. Nevertheless, under minimum percentage of opening (0%), temperature variables were higher: 82.42 °C, 83.16 °C, and 82.05 °C. In parallel, the monitored system received the heat requirements from resistance, being coordinates as a result. Therefore, comprehensive evaluation of the mentioned transmitters, FCV0101, and resistance was suitable for the monitoring purposes.

**Figure 20:**
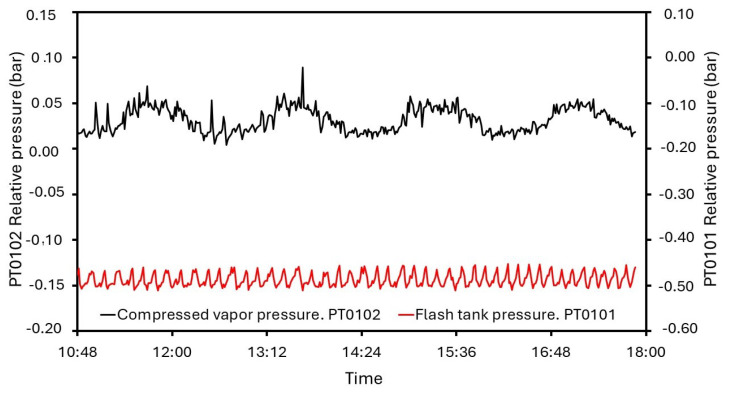
Measurements from PT0101 and PT0102 for steady state.

**Figure 21:**
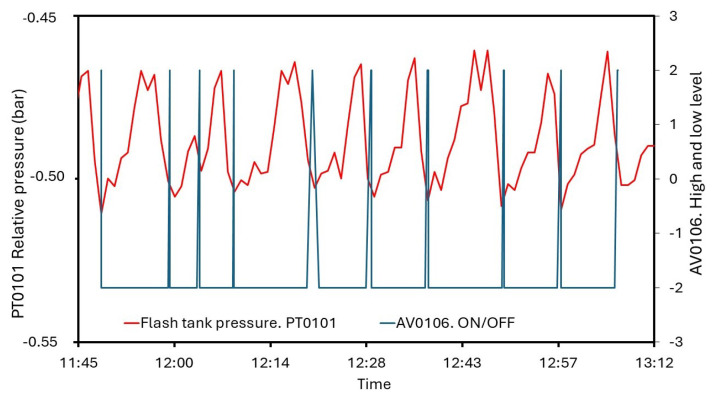
AV0106 performance depending on measurements of PT0101.

Generally, closed systems which involved thermal and mechanical agents in the desalination process make detailed measurements and the control of pressure mandatory. Some undesired variations can modify the boiling point, and the efficiency will not be optimal, so pressure sensing and monitoring is developed and analyzed. In Figure 20, the transmitters’ evolution in the flash tank and at the compressor outlet is represented. The T0102 relative pressure stays around −0.48 bar and is controlled by the system. AV0106 ensures higher values than −0.50 bar. However, a passing peak of −0.51 bar is found, detecting a delay in response time. In Figure 21, the readings of PT0101 and AV0106 are represented. Also, pressure at the compressor outlet is controlled by AV0110 with a set point of 0.05 bar. In general, pressure remains about this value, but the triggering is slower, and the maximum reading is 0.089 bar. Commonly, the behavior of the measuring and control system for pressure is appropriate but valve delays lead to reach some values which do not match with the set points.

### 3.3. Mass and Energy Balance

**Figure 22:**
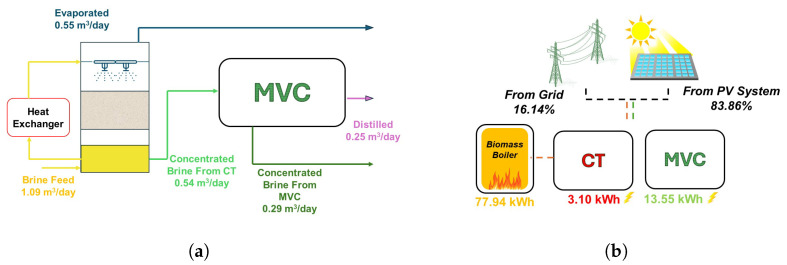
Balance data for the hybrid technology. (**a**) Mass balance. (**b**) Energy balance: electrical and thermal consumption.

Future works will include a comprehensive balance of energy and mass with theoretical and experimental data. A preliminary result is shown to deliver more thorough information about the functionality of the technology. The system has a capacity of 1.09 m^3^/day, working 8 h on average as illustrated in Figure 22. Streams are shown in different colors due to salinity and physical state differences. The percentage of freshwater recovery is 24%, evaporating 50% in the CT. Rejected and more densely concentrated brine represents 26%, and as a consequence, the waste is reduced 74%, approximately. Principally, renewable sources are the motor of the system as shown in Figure 22. Streams are shown in different colors due to salinity and physical state differences. Electrical consumption is measured by an installed network analyzer. An average of 13.55 kWh is obtained for MVC, while CT has a lower value of 3.10 kWh. Mainly, this energy comes from sun representing 83.86%. Heat energy is required to warm the brine. CT performance is satisfactory as the temperature increases, so a biomass boiler is implemented. The power is 100 kW and according to the pellet consumption, 77.94 kWh is the estimated heat energy.

## 4. Conclusions

The enhancing operational arranging an accurate solution for the monitoring and sensing was developed and evaluated during the study. In the cooling tower, temperature and humidity sensors were installed. The main water at the boiler outlet swept across a spectrum of measurements from ambient conditions to 70 °C. Thus, the device is able to determine any temperature for the workplace settings. Also, PT1000 seemed to be suitable for brine readouts. The illustrated scenario in Section 3 showed a range from 35 to 21 °C, but with more heating hours, the brine rose 60 °C. Moreover, the humidity sensor exhibited a fitting behavior both for relative humidity levels around 90% and for the lower records of 40% approximately. As well, the sensor worked in a common temperature range for the location, specifically, air ranged between 25 and 13 °C. Finally, the flow meter was examined at the inferior bound of the interval (12.70 to 19.90 L/min), showing a good response. Overall, the proposal sensing has swift adaptability and the global solution has a broad scope to future thermal works.

The developed control system in MVC produced positive results. Provided measurements from the TT0104 sensor–transmitter were integrated alongside the management device; thus, two programmable temperature ranges were tested for the external energy source. The selected interval was 82 to 84 °C. In general, the drive of the resistance was satisfactory, compared with the TT0104 readings, despite the fluctuations outside the set point caused by the inertia phenomena and the possible delays in the turning on/off.

As was mentioned, pressure is a variable of interest. Both installed sensor–transmitters in MVC, PT0101 and PT0102 were measuring and sending information to the regulation system, successfully. Devices were configured to maintain T0102 above −0.5 bar, but PT0101, recording data each minute, registered a minimum peak of −0.51 bar. Compressed vapor showed the highest pressure values. For security, PT0102 had to register a pressure under 0.05 bar. An average of 0.031 was found, even though there existed a measured 0.089 bar. In light of these results, the monitoring and control system exerted an accurate action over the pressure variable, visualizing punctual records out of range.

In addition, the other important variable of interest is temperature. Sensor–transmitters located at critical points were evaluated. All of them recorded data each minute without any anomaly. As noted, the measuring of TT0104 had a significant role because the transmission of information controlled the startup of the resistance. The device exhibited an appropriate behavior, showing an interval from 81.88 to 84.19 °C, approximately. TT0101, 2, 3 and 5 were working at the same range, between 81.86 and 84.29 °C. The last identical sensors at the compressor outlet displayed the highest values for the vapor stream, reaching 100 °C.

Two pathways for the heat requirements are considered in this work: the resistance and the vapor recirculation from FCV0101. The programmable valve demonstrated proper performance. The gathered results were analyzed in terms of percentage. The maximum collected rate of the opening was automated during resistance turning off; for this period, FCV0101 allowed the vapor recirculation in 50% at least, while its actuation gradually diminished until the startup of the resistance. The implemented communication tools were tested adequately. Remote control was executed successfully, and the equipment could be disconnected from outside the desalination plant. Also, the HMI panel connected to Siemens Switch was useful to have information about the process progress, receiving it from PLC. Different problems with the synchronization of the recorded data were found in the case study evaluation. While temperature and pressure in MVC were saved each minute, the elements actuation (pumps, valves, and resistance) was saved in real time, due to memory capacity constraints. Future works lead to a programmable file, where relevant variables and the operation elements offer aligned information. Despite the high salinity values, CT could reach 50 mS/cm and MVC 70 mS/cm. The sensors and the equipment showed no anomalies under these conditions. The combination of thermal (CT) and mechanical (MVC) processes reduces the brine rejection to 26% in relation to the total brine input, producing a concentrate brine from 50 mS/cm to higher than 200 mS/cm at outlet of the combined system. The standalone CT and MVC result in a lower diminution of the inlet. CT can evaporate the 50%, while MVC distillates the 46%, so the hybrid technology increases the treatment capacity. The control system described in this article is tuned for safe and stable operation and is adjusted for different startup processes depending on the environmental conditions encountered throughout the winter months in the test area.

As a consequence of the green energies implementation, renewable sources produce 83.86% of the electrical consumption. The highest value was registered for MVC at 13.55 kWh, and CT is more conservative in terms of power at 3.10 kWh. Brine is heated by the heat exchanger, and energy comes from the biomass boiler, using 77.94 kWh. Therefore, the hybrid technology is almost entirely powered by clean energy.

Nowadays, the development of technologies has led to improved control of monitoring systems in industrial environments. The optimization according to new algorithms or the deployment of AI will result in a more robust and comprehensive system. Upcoming studies will propose the integration of emerging innovations in desalination. Future works will be planned following the energetic and thermal line. A study of the consumption peaks with the resistance turning on and off is a research purpose, as well as the search for optimal control parameters for both systems, CT and MVC, depending on the environmental conditions and the brine conditions at the inlet. As always, the aim is minimizing the energy consumption required for each system.

## Figures and Tables

**Table 1:** Temperature and humidity sensors: technical data.

PT1000 of Four Wires: Almeno-ZA9030	Humidity, Temperature and Pressure Sensor: Almeno-FHAD 46-C41AL05	Flow Meter: FVA645GV200QT5
Accuracy: 0.07 K	Measurement method: Capacitive and barometric	Measurement method: Pressure pulsation. Kármán vortex street
Measuring range: −200 to 400 °C	Measuring ranges: 5 to 98% relative humidity (HR), −40 to +85 °C and 700 to 1100 mbar, respectively	Measuring range: 10 to 200 L/min
Resolution: 0.01 °C	Resolution: Humidity: 0.1% RH. Temperature: 0.1 K. Pressure: 0.1 mbar	Resolution: 0.1 L/min
Output signal: 4–20 mA. Analog/digital conversion in data logger	Integrated A/D converter	Output signal: 2 × 0.5 to 3.5 V (4.1 V)

**Table 2:** Almeno 2890-9 data logger technical data.

Precision class	AA IEC 60751
Inputs	9 input sockets for connecting sensors
Outputs	2 output communication sockets. Connection with a digital interface by USB, Ethernet, WLAN, RS232, wireless with Bluetooth
Measuring rate	100, 50, 10 and 2.5 mops
Internal memory	EEPROM 100,000 measured values
Measurements/s	2.5 to 100 measurements per second

**Table 3:** Technical data of MVC instrumentation.

Instrument	Type	Model	General Features	Function
Sensors and transmitters, from TT0101 to TT0107	PT1000 for liquid and gases	TD2251–IFM	−50 to 150 °C. Analog signal of current: 4–20 mA	Data measuring and transmission. Readings of the sensors 3, 4, 6 and 7 influences on control mechanism.
Valves. AV010–6, 7, 10, 11, 12, 13 and 14	Angle seat valve. Pneumatic	SN NC Element 2100–Bürket	Flow rate: from 4.8 m^3^/h to 140 m^3^/h. Pressure control: from 2.5 to 10 bar. Working fluid: air, neutral gases or vapor	AV0110: Pressure control in the output of compressor. It opens at 0.05 bar, when PT0102 is 0.15 bar, compressor shuts down at 0.15 bar. AV0111: Venting for volatiles in T–0104. Programmed to open five seconds each five second time interval. AV0113: Programmed to inject distillate before the compressor input.
Valves. From AV0101 to AV0105	Solenoid valve with pneumatic actuator	PP10S SE NC + CFC-5601 + EV–500.024C–SAFI	Nominal flow rate: 1730 L/min. Pressure: from 2 to 8 bar.	Feed valves: from AV0101 to AV0104. Programmed according to the level sensors. Concentrated brine, AV0105, programmed by time.
AV0108 and AV0109	Ball valve with pneumatic actuator	GNP–44–S5/F5/E14–Genebre	Air working pressure: from 2 to 8 bar. Ball valve of 1–inch	Control of condensates from compressor.
FCV0101	Pneumatic control valve type 2301 with programmable head	8696–Bürkert.	Measurements of valve position. Programming of valve adjustment with 4–20 mA signal	Control of vapor recirculation into flash tank.
PT0101, pressure transmitter		PX3524–IFM	Measurement range: −1 to 10 bar. Analog output, 4–20 mA.	Measuring and transmission of data
PT0102, pressure transmitter		PI2795–IFM	Measurement range: −1 to 4 bar. Analog output, 4–20 mA.	Measuring and transmission of data

## Data Availability

The data presented in this study are available on request from the corresponding author.

## Abbreviations

The following abbreviations are used in this manuscript:

BGW	Brackish groundwater
CSP	Concentrated solar power
CT	Cooling tower
CTW	Cooling tower water
ED	Electrodialysis
ME-MVC	Multi-effect mechanical vapor compression
MED	Multi-effect desalination systems
MVC	Mechanical vapor compression
PLC	Programmable logic controller
PVT-PV	Photovoltaic/thermal-photovoltaic
RES	Renewable energy sources
RO	Reverse osmosis
SGW	Saline groundwater
TDS	Total dissolved solids
ZLD	Zero Liquid Discharge
